# Comparing Kidney Transplant Rates and Outcomes Among Adults With and Without Intellectual and Developmental Disabilities

**DOI:** 10.1001/jamasurg.2022.7753

**Published:** 2023-02-15

**Authors:** Brittany N. Hand, J. Madison Hyer, Austin Schenk, Alex Coyne, Daniel Gilmore, Lauren Wang, Aslam Ejaz

**Affiliations:** 1 School of Health and Rehabilitation Sciences, The Ohio State University, Columbus; 2 Center for Biostatistics, The Ohio State University, Columbus; 3 Department of Surgery, The Ohio State University, Columbus; 4 Timothy Freeman Center for Intellectual and Developmental Disabilities, University of Cincinnati, Cincinnati, Ohio

## Abstract

**Importance:**

Improving equity in organ transplant access for people with intellectual and developmental disabilities (IDD) is a topic of social discourse in mainstream media, state legislation, and national legislation. However, few studies have compared evaluation rates, transplant rates, and outcomes among adults with and without IDD.

**Objective:**

To compare rates of kidney transplant and transplant-specific outcomes between propensity–score matched groups of adults with end-stage kidney disease (ESKD [also referred to as end-stage renal disease (ESRD)]) with and without co-occurring IDD.

**Design, Setting, and Participants:**

This retrospective cohort study included all Medicare inpatient and outpatient standard analytical files from 2013 through 2020. A total of 1 413 655 adult Medicare beneficiaries with ESKD were identified. Propensity–score matching was used to balance cohorts based on age, sex, race, follow-up duration, and Charlson Comorbidity Index. The matched cohorts consisted of 21 384 adults with ESKD (10 692 of whom had IDD) and 1258 kidney transplant recipients (629 of whom had IDD). Data were analyzed between June 1, 2022, and August 1, 2022.

**Exposure:**

IDD.

**Main Outcomes and Measures:**

Evaluation for kidney transplant, receipt of kidney transplant, perioperative complications, readmission, mortality, graft rejection, and graft failure.

**Results:**

Of the 21 384 propensity–score matched adults with ESKD, the median (IQR) age was 55 (43-65) years, 39.2% were male, 27.4% were Black, 64.1% were White, and 8.5% identified as another race or ethnicity. After propensity score matching within the ESKD cohort, 633 patients with IDD (5.9%) received a kidney transplant compared with 1367 of adults without IDD (12.8%). Adults with IDD were 54% less likely than matched peers without IDD to be evaluated for transplant (odds ratio, 0.46; 95% CI, 0.43-0.50) and 62% less likely to receive a kidney transplant (odds ratio, 0.38; 95% CI, 0.34-0.42). Among matched cohorts of kidney transplant recipients, rates of perioperative complications, readmission, and graft failure were similar for adults with and without IDD.

**Conclusions and Relevance:**

Using the largest cohort of adult kidney transplant recipients with IDD to date, the study team found that rates of evaluation and transplant were lower despite yielding equivalent outcomes. These data support consideration of adults with IDD for kidney transplant and underscore the urgent need for antidiscrimination initiatives to promote the receipt of equitable care for this population.

## Key Points

**Question:**

How do rates of kidney transplant and transplant outcomes differ for adults with vs adults without intellectual and developmental disabilities (IDD)?

**Findings:**

In this cohort study, adults with IDD were 54% less likely to be evaluated for and 62% less likely to receive a kidney transplant than adults without IDD. However, among those who received a kidney transplant, postoperative outcomes were similar for adults with and without IDD.

**Meaning:**

These data suggest the IDD should not categorically disqualify adults from transplant and underscore the urgent need for antidiscrimination initiatives to promote the receipt of equitable care for this population.

## Teaser

This study compares rates of kidney transplant and transplant-specific outcomes between propensity-score matched groups of adults with end-stage kidney disease with and without co-occurring intellectual and developmental disabilities.

## Introduction

The increasing demand for organ transplant is outpacing the current supply of donor organs,^1^ requiring transplant centers to prioritize which patients to put on transplant wait lists. Despite existing legislation, such as the Americans With Disabilities Act, Rehabilitation Act, and the Affordable Care Act, that prohibits discrimination against people with disabilities, people with intellectual and developmental disabilities (IDD) seeking solid organ transplants continue to be discriminated against.^2,3,4^ Indeed, most surgeons (57% to 89%) consider moderate to profound intellectual disability a contraindication to receiving a transplant.^4^

The population of people with IDD is heterogeneous, representing a broad range of independence in everyday activities, cognitive functioning, and medical complexity. Despite the high degree of variability within this population, one commonly cited reason for excluding people with IDD from transplant wait lists is concern that they may have difficulty following posttransplant care, putting them at greater risk of perioperative complications, mortality, or graft failure.^2^ However, an emerging body of literature, primarily using pediatric cohorts, does not support this notion. Most pediatric studies have found similar transplant outcomes for children with and without IDD,^5,6,7,8,9,10^ while only a few studies have found worse graft or patient survival among those with IDD.^11,12,13^ As a result, many have advocated against the absolute exclusion of individuals with IDD from transplant wait lists^2,14,15,16^ and some states have passed legislation specifically prohibiting discrimination against people with disabilities in need of organ transplant.^2^ However, discrimination continues to occur in these states,^17^ making these laws a “symbolic gesture rather than a genuine shield against discrimination.”^18^

As a result of this observed discrimination against people with IDD, there is growing national momentum to enact and enforce policies to improve equitable access to organ transplant for people with IDD and other disabilities. Indeed, this topic was identified as a key highlight of the 2022 American Medical Association conference^19^ and the US Department of Health and Human Services is currently seeking public comments on how to improve equity and reduce disparities in organ transplant.^20^ At the congressional level, a new US House bill is under consideration that would prohibit surgeons, hospitals, transplant centers, and other health care professionals from denying access to organ transplants to someone because they have a disability. This proposed federal legislation has launched the topic of equity in organ transplant into mainstream media and social dialogue.^21,22,23^ However, studies using adult cohorts to inform evidence-based policy making initiatives are lacking.

In this study, we aim to add to the growing body of literature comparing transplant outcomes among adults with and without IDD. While most studies to date have focused on pediatric cohorts, our study offers a unique contribution to the literature by analyzing data from a large national cohort of adults with end-stage kidney disease (ESKD [also referred to as end-stage renal disease (ESRD)]) with and without IDD. Specifically, we sought to compare rates of evaluation, rates of transplant, and transplant outcomes.

## Methods

### Data Source

Data used for this study were derived from Medicare Standard Analytical Files for the years 2013 through 2020, which include limited data set information on 100% of Medicare beneficiaries for these years. Deidentified, beneficiary-level health care claims data for inpatient and outpatient records were used. Outpatient records contained medical billing claims from institutional outpatient facilities (ie, hospital outpatient departments, rural health clinics, kidney dialysis facilities, outpatient rehabilitation facilities, Federally Qualified Health Centers, and community mental health centers) but do not include professional service claims from noninstitutional professional facilities (ie, physicians, physician assistants, clinical social workers, or nurse practitioners).

### Study Population and Cohort Selection

Medicare beneficiaries with ESKD were included if they: (1) were 18 years of age or older and (2) had at least 1 inpatient or outpatient encounter with a diagnosis of ESKD at any time during January 1, 2012, to December 31, 2020. ESKD was identified using *International Classification of Diseases, Ninth Revision *(*ICD-9*) code 585.6 and *International Statistical Classification of Diseases and Related Health Problems, Tenth Revision * (*ICD-10*) codes Z99.2 or N18.6. Adults with IDD were identified based on at least 1 encounter with *ICD-9* or *ICD-10* codes for intellectual disability (*ICD-9*: 317 to 319; ICD-10: F70 to F79), pervasive developmental disorders (*ICD-9*: 299.X; *ICD-10*: F84.X), cerebral palsy (*ICD-9*: 343.X; *ICD-10*: G80.X), or Down syndrome (*ICD-9*: 758.0, *ICD-10*: Q90).

Once eligible beneficiaries were identified, 2 rounds of propensity score matching were performed: 1 for the cohort of all adults with ESKD (and thus potentially eligible for kidney transplant) and 1 among only those who received a kidney transplant. Propensity score matching was conducted using logistic regression to predict the probability that, given a set of observed covariates, a beneficiary has IDD.^24^ This model resulted in a propensity score derived from the beneficiary’s age, sex, race, duration of follow-up, and Charlson Comorbidity Index (CCI). CCI is a continuous predictor of mortality risk and was used in this study as defined by Quan and colleagues^25^ (both *ICD-9* and *ICD-10* versions depending on the year of data), which captures 17 types of health conditions (eg, myocardial infarction, congestive heart failure, cerebrovascular disease, etc). Matching was performed without replacement using a greedy nearest available neighbor algorithm.^26^ The quality of the match was confirmed with standardized mean differences on observed variables less than 0.25 and graphical displays of propensity score distributions in the unmatched and matched cohorts.^27^ The institutional review board of our institution reviewed this study and determined it to be exempt due to the use of limited data sets.

### Outcome Measures

Outcomes examined in this study were evaluation by a transplant surgeon, receipt of kidney transplant, and postoperative outcomes. Postoperative outcomes included any perioperative complication, 90-day all-cause readmission, 1-year mortality, any graft failure within 1 year, and graft rejection within 1 year. Evaluation by a transplant surgeon was identified by the patient having a medical encounter with a health care professional who performed at least 1 kidney transplant during 2013 through 2020. Kidney transplant was identified by *ICD-9* procedure code 55.6X, *ICD-10* procedure codes 0TY0 or 0TY1, or *Current Procedural Terminology *code 53060. Perioperative complications were identified by an encounter with an *ICD-9* or *ICD-10* diagnosis of pulmonary failure, pneumonia, myocardial infarction, deep venous thrombosis, pulmonary embolism, acute kidney failure, surgical site infection, gastrointestinal bleeding, or postoperative hemorrhage within 30 days of transplant.^28,29,30^ Mortality data were derived based on the date of death in Medicare Standard Analytical Files. Graft failure was identified by any incidence of dialysis 90 to 365 days posttransplant using revenue center codes 0820, 0821, 0824, 0829 to 0831, 0834, or 0839. Graft rejection was identified by *ICD-10* code T86.11; given that there is not a corresponding *ICD-9* code with an equal degree of specificity, we only examined graft rejection among those who had their kidney transplant in October 2015 or later.

### Statistical Analysis

Descriptive statistics were presented as median (25th to 75th percentiles [IQR] and frequency relative frequency, percentage) for continuous and categorical measures, respectively. To assess whether patients with ESKD receive transplants at different rates based on whether or not they have IDD, a multivariable logistic regression model with a log (follow-up months) offset was used. Furthermore, among patients who received a transplant, multivariable logistic regression models were used to determine whether patients with IDD have different postoperative outcomes than those without IDD. The models controlled for sex, age, and CCI. All analyses were performed using SAS version 9.4 (SAS Institute). Statistical significance (2-sided) was assessed at α = .05.

## Results

### Cohort Characteristics

Characteristics of the unmatched cohorts are provided in the eTable in the Supplement. The propensity score–matched cohort of patients with ESKD consisted of 10 692 adults with IDD and 10 692 without IDD (Table 1). The median age was 55 years and approximately 39% were male. The most common IDD diagnosis was other/unspecified intellectual disability (61.4% of IDD cohort) followed by cerebral palsy (21.5% of IDD cohort).

**Table 1.** Characteristics of Propensity Score–Matched Cohorts

Characteristic	Adults with end-stage kidney disease			Kidney transplant recipients		
	Total (n = 21 384)	PC (n = 10 692)	IDD (n = 10 692)	Total (n = 1258)	PC (n = 629)	IDD (n = 629)^a^
Age, median (IQR), y	55 (43-65)	55 (43-65)	55 (43-65)	37 (29-49)	38 (29-49)	37 (29-49)
Sex, No. (%)						
Female	12 994 (60.8)	6497 (60.8)	6497 (60.8)	840 (66.8)	420 (66.8)	420 (66.8)
Male	8390 (39.2)	4195 (39.2)	4195 (39.2)	418 (33.2)	209 (33.2)	209 (33.2)
Race,^b^ No. (%)						
Black	5864 (27.4)	2932 (27.4)	2932 (27.4)	276 (21.9)	138 (21.9)	138 (21.9)
White	13 702 (64.1)	6851 (64.1)	6851 (64.1)	812 (64.5)	406 (64.5)	406 (64.5)
Other^c^ or unknown	1818 (8.5)	909 (8.5)	909 (8.5)	170 (13.5)	85 (13.5)	85 (13.5)
CCI, median (IQR)	3 (1-5)	3 (1-5)	3 (1-5)	2 (1-4)	2 (1-4)	2 (1-4)
IDD diagnosis^d^						
Cerebral palsy	2293 (10.7)	NA	2293 (21.5)	131 (10.4)	NA	131 (20.8)
Down syndrome	975 (4.6)	NA	975 (9.1)	74 (5.9)	NA	74 (11.8)
Intellectual disability						
Mild	2119 (9.9)	NA	2119 (19.8)	119 (9.5)	NA	119 (18.9)
Moderate	811 (3.8)	NA	811 (7.6)	34 (2.7)	NA	34 (5.4)
Profound/severe	857 (4.0)	NA	857 (8.0)	47 (3.7)	NA	47 (7.5)
Other/unspecified	6563 (30.1)	NA	6563 (61.4)	395 (31.4)	NA	395 (62.8)
Pervasive developmental disorders						
Autism	1037 (4.7)	NA	1037 (9.7)	43 (3.4)	NA	43 (6.8)
Other	311 (1.4)	NA	311 (2.9)	25 (2.0)	NA	25 (4.0)
Evaluation by surgeon	5416 (25.3)	3271 (30.6)	2125 (19.9)	NA	NA	NA
Transplant received	2000 (9.4)	1367 (12.8)	633 (5.9)^a^	NA	NA	NA
Perioperative complication	NA	NA	NA	384 (30.5)	185 (29.4)	199 (31.6)
90-d Readmission	NA	NA	NA	503 (40.0)	244 (38.8)	259 (41.2)
1-y Mortality	NA	NA	NA	26 (2.1)	NA^e^	NA^e^
Graft rejection within 1 y	NA	NA	NA	97 (7.7)	47 (7.5)	50 (8.0)
Graft failure within 1 y	NA	NA	NA	22 (1.8)	NA^e^	NA^e^

Abbreviations: CCI, Charlson Comorbidity Index; IDD, intellectual or developmental disabilities; NA, not applicable; PC, population comparison.

^a^ Three kidney transplant recipients with IDD were unable to be matched.

^b^ Based on Centers for Medicare and Medicaid Services data.

^c^ Includes Asian, Hispanic, North American Native, and other.

^d^ These diagnoses are not mutually exclusive, so a single individual could be listed in multiple rows.

^e^ Data cannot be presented due to cell size restrictions in the data use agreement.

The propensity score–matched cohort of kidney transplant recipients consisted of 629 adults with IDD and 629 adults without IDD (Table 1). The median age was 37 years and approximately 33% were male. Approximately 45% of the IDD cohort had other/unspecified intellectual disability and 22.7% had cerebral palsy.

### Evaluation by Transplant Surgeon

Within the matched ESKD cohort, 2125 adults with IDD (19.9%) were evaluated by a transplant surgeon in contrast with 3271 adults without IDD (30.6%) (Table 1). Multivariable analysis revealed adults with IDD had 54% lower odds (95% CI, 0.43-0.50) of being evaluated by a transplant surgeon than those without IDD (Table 2).

**Table 2.** Odds of Being Evaluated by a Transplant Surgeon and Receiving a Transplant Among Adults With End-stage Kidney Disease With and Without Intellectual or Developmental Disability

Outcome	Odds ratio (95% CI)
Evaluation	0.46 (0.43-0.50)
Receipt of transplant^a^	0.38 (0.34-0.42)
Receipt of transplant^b^	0.49 (0.43-0.55)

^a^ Among all patients with a diagnosis of end-stage kidney disease.

^b^ Among the subset of patients who were evaluated by a transplant surgeon.

### Receipt of Transplant

Within the matched ESKD cohort, 633 adults with IDD (5.9%) received a transplant in contrast with 1367 adults without IDD (12.8%) (Table 1). Multivariable analysis revealed adults with IDD had 62% lower odds (95% CI, 0.34-0.42) of receiving a transplant than those without IDD (Table 2). Furthermore, among the subset of patients who were evaluated by a transplant surgeon, 624 adults with IDD (29.4%) and 1357 adults without IDD (41.2%) received a transplant. Multivariable analysis revealed that, among those who were evaluated by a surgeon, adults with IDD had 51% lower odds (95% CI, 0.43-0.55) of receiving a transplant than those without IDD (Table 2).

### Postoperative Outcomes

Within the matched transplant cohort, 384 patients (30.5%) experienced a perioperative complication, 503 patients had a 90-day readmission (40.0%), 97 experienced graft rejection within 1 year (7.7%), and 22 experienced graft failure within 1 year (1.8%) (Table 1). Rates of adverse postoperative outcomes did not differ between adults with and without IDD. Specifically, patients without IDD had similar rates of perioperative morbidity (n = 185 [29.4%] vs n = 199 [31.6%]), 90-day readmission (n = 244 [38.8%] vs n = 259 [41.2%]), and 1-year graft failure (numbers censored due to cell size restrictions) compared with patients with IDD. Multivariable analysis revealed IDD was not a risk factor for perioperative complications, 90-day readmission, or 1-year graft rejection (Table 3).

**Table 3.** Perioperative, 90-Day, and 1-Year Outcomes Among Kidney Transplant Recipients With and Without Intellectual or Developmental Disability

Outcome	Odds ratio (95% CI)
Perioperative complication	1.09 (0.85-1.39)
90-d Readmission	1.13 (0.89-1.46)
1-y Mortality	NA^a^
Graft rejection within 1 y	1.05 (0.69-1.64)
Graft failure within 1 y	NA^a^

Abbreviation: NA, not applicable.

^a^ Multivariable output not reported due to concerns about estimate stability due to rarity of event.

## Discussion

Using a propensity score–matched comparison group, our findings show that adults with IDD were significantly less likely to be evaluated for or receive a kidney transplant than adults without IDD. Yet, among those who did receive a kidney transplant, perioperative, 90-day, and 1-year outcomes were similar for adults with and without IDD. This study adds to a growing US national discourse around equal access to organ transplant for people with IDD. We recognize that organ transplant is a lifelong care-intensive undertaking and that, like peers without IDD, some adults with IDD may not be strong candidates for transplant. However, adults with IDD deserve (and legally have a right to) equal access to evaluation and full holistic consideration as to whether they would be good transplant candidates. Ultimately, our results bolster the body of evidence supporting full consideration of adults with IDD for kidney transplant and the urgent need for antidiscrimination initiatives.

Consistent with prior studies, our findings indicate people with IDD have similar transplant outcomes to the general population. Specifically, we found that the incidence of perioperative complications, 90-day readmission, and 1-year graft failure was similar between patients with and without IDD. These findings are consistent with prior studies; however, previous studies on solid organ transplant outcomes among individuals with IDD have largely focused on pediatric cohorts.^5,7,8,9,10^ Indeed, only a handful of studies to date have included adults with IDD.^6,31,32,33^ While these studies consistently found that transplant outcomes are similar among adults with and without IDD, they were limited by small sample sizes of adult transplant recipients with IDD (3 to 5 patients per study) and were often restricted to a single transplant center or geographic region. Our study makes a unique and valuable contribution to the literature on this topic by analyzing the largest US national cohort of adults with IDD who received kidney transplants. Furthermore, as all individuals in the US with ESKD are eligible for Medicare, our cohort provides comprehensive information about the prevalence of receiving a kidney transplant among adults with and without IDD. Taken together, our results indicated that IDD is not a risk factor for worse outcomes following transplant, despite misconceptions about these patients.^2^

This study has important implications for antidiscrimination education materials and policies. First, there is an urgent need for antidiscrimination continuing medical education materials^19^ to (1) combat the misconception that IDD is a contraindication to receiving a transplant^4^ and (2) encourage health care professionals to examine how their implicit biases toward people with IDD influence their decision making in the organ transplant process.^34^ Education initiatives also need to ensure that health care professionals and health care systems are aware of and comply with existing legislation that prohibits discrimination on the basis of disability in the organ transplant process.^17^ Second, there is a need for policy to improve transparency and reduce disparities in the transplant allocation system.^35^ If enacted into law, US House bill 1235 may be a promising step forward in this area by detailing specific conditions under which an individual’s disability may or may not be taken into consideration in the organ transplant process. The findings of this study add to the base of evidence supporting policies like US House bill 1235 and the development of processes for monitoring adherence to antidiscrimination legislation.

Our data suggest discrimination against adults with IDD in the organ transplant process may begin as early as referral of patients with ESKD to transplant surgeons for evaluation. In our study, adults with IDD were 54% less likely than peers without IDD to be evaluated by a transplant surgeon. Therefore, antidiscrimination initiatives should include focusing on primary care professionals, nephrologists, and dialysis clinics to ensure equity in referral rates. Additionally, adults with IDD may be less likely to be listed for or receive a transplant, which would suggest a need to scrutinize transplant center practices. In our study, adults with IDD who were evaluated by a transplant surgeon were 51% less likely than their peers to receive a kidney transplant. However, the Medicare database does not contain information about which patients with ESKD were or were not listed for transplants nor the reason behind such a decision; these data also do not indicate if patients listed for transplants were delisted or died prior to transplant. Rather, our data reflect the pool of all adults in the US with ESKD and indicate which of those adults received a transplant. Therefore, future studies would be needed to further examine which stage(s) in the organ transplant process are the most important to evaluate for possible discrimination and target for antidiscrimination initiatives as needed.

We recognize it is likely that adults with IDD in our sample who received a kidney transplant were selected for listing by the transplant center due to a high likelihood of successful outcomes. Transplant centers likely selected adults with IDD who, either independently or with support, can fulfill transplant center protocols. This may result in discrimination against adults with IDD with more significant support needs. This may indicate that transplant centers are unable or unwilling to provide the additional pretransplant and posttransplant resources that some adults with IDD may require. Previous studies have shown that preoperative preparation and education, as well as ongoing follow-up support, have improved outcomes of adults with IDD for other surgical procedures (ie, bariatric surgery).^36,37,38^ This could also be a promising approach for adults with IDD who are seeking organ transplant, but further work is needed to evaluate the impacts of prolonged education and support on transplant outcomes in this population.

### Limitations

There are several limitations for this study that should be considered when interpreting these findings. First, it is possible that some adults with IDD were excluded from the IDD cohort if they did not have an observed encounter during 2013 through 2020 with an IDD diagnosis. This may have resulted in some adults with IDD incorrectly being included in the control cohort of adults without IDD. Second, Medicare data do not contain information about whether the kidney donors were deceased or living. As a result, we could not control for this in our analysis or examine the extent to which differences in donor type may have affected our findings. Third, Medicare data do not contain psychosocial variables indicative of transplant appropriateness (eg, Stanford Integrated Psychosocial Assessment for Transplant^39^) and likelihood of postoperative care adherence.^40,41,42^ Future studies are needed to determine how to best evaluate psychosocial factors linked with transplant outcomes in adults with IDD and the effect of readiness for a transplant, available support systems, and other psychosocial factors on rates of transplant among this population. Fourth, examining postoperative adequacy of care and treatment adherence was beyond the scope of this study, but is an important consideration for future work. Lastly, we were unable to determine how many beneficiaries (with or without IDD) chose not to move forward with the transplant process, despite being offered the opportunity to do so. Among the general population, prior studies have documented concerns about finances, inequity in the transplant selection process, and passing the medical tests required for transplant listing influence patients’ decisions about whether to pursue transplant.^43,44^ Some caregivers of adults with ESKD may also discourage transplant due to the believe that it would take a larger toll on their own mental and physical well-being than supporting someone on hemodialysis, although current literature does not support this.^45^ To our knowledge, no studies have evaluated factors affecting the selection of kidney replacement therapy specifically among adults with IDD and their caregivers; this may be an important direction for future work.

## Conclusions

There is growing national momentum to enact and enforce policies to improve equitable access to organ transplant for people with IDD and other disabilities. Like peers without IDD, some adults with IDD may not be strong candidates for transplant. However, adults with IDD deserve (and legally have a right to) equal access to evaluation and full holistic consideration as to whether they would be good transplant candidates. Our findings show that adults with IDD were significantly less likely to be evaluated for or receive kidney transplants than propensity score–matched peers without IDD. Using the largest US cohort of adult transplant recipients with IDD to date, we found that perioperative, 90-day, and 1-year kidney transplant outcomes were similar for adults with and without IDD. Ultimately, these results support kidney transplant among adults with IDD and underscore the urgent need for antidiscrimination initiatives to promote the receipt of equitable care for this population.

## Notes

Supplement. eTable 1. Characteristics of unmatched cohorts
