# Supplementary material for: Comparing Kidney Transplant Rates and Outcomes Among Adults With and Without Intellectual and Developmental Disabilities
Source: JAMA Surg. 2023 Feb 15;158(4):386–92. doi: 10.1001/jamasurg.2022.7753 (PMC9932938; doi:10.1001/jamasurg.2022.7753)
Supplement: Supplement. — eTable 1. Characteristics of unmatched cohorts [file jamasurg-e227753-s001.pdf]

Supplemental Online Content

Hand BN, Hyer JM, Schenk A, et al. Comparing kidney transplant rates and outcomes among adults with and without intellectual and developmental disabilities. *JAMA Surg.* Published online February 15, 2023. doi:10.1001/jamasurg.2022.7753

eTable 1. Characteristics of unmatched cohorts

This supplemental material has been provided by the authors to give readers additional information about their work.

© 2023 Hand BN et al. *JAMA Surgery.*

eTable 1: Characteristics of unmatched cohorts

	Adults with end-stage kidney disease				Kidney transplant recipients		
	Total<br>N = 1,413,655	PC<br>N = 1,402,960	IDD<br>N = 10,695		Total<br>N = 95,519	PC<br>N = 94,886	IDD<br>N = 633
Age in years, median (IQR)	66 (56, 76)	67 (56, 76)	55 (43, 65)		52 (41, 61)	52 (41, 61)	37 (28, 49)
Male, n (%)	620,556 (43.9)	616,252 (43.9)	4,304 (40.2)		36,900 (38.6)	36,690 (38.7)	210 (33.2)
Race, n (%)							
Black	386,831 (27.4)	383,898 (27.4)	2,933 (27.4)		29,211 (30.6)	29,073 (30.6)	138 (21.8)
White	825,602 (58.4)	818,750 (58.4)	6,852 (64.1)		44,776 (46.9)	44,366 (46.8)	410 (64.8)
Other or unknown	201,222 (14.2)	200,312 (14.3)	910 (8.5)		21,532 (22.5)	21,447 (22.6)	85 (13.4)
CCI, median (IQR)	4 (2, 5)	4 (2, 5)	3 (1, 5)		2 (1, 4)	2 (1, 4)	2 (1, 4)
IDD diagnosis, n (%)[b]							
Cerebral palsy	2,406 (0.2)	--	2,406 (21.8)		144 (0.2)	--	144 (22.8)
Down syndrome	1,011 (0.1)	--	1,011 (9.2)		69 (0.1)	--	69 (10.9)
Intellectual disability							
Mild	2,147 (0.2)	--	2,147 (19.5)		118 (0.1)	--	118 (18.6)
Moderate	824 (0.1)	--	824 (7.5)		38 (<0.1)	--	38 (6.0)
Profound/severe	870 (0.1)	--	870 (7.9)		17 (<0.1)	--	17 (2.7)
Other/ unspecified	6,637 (0.5)	--	6,637 (60.2)		286 (0.3)	--	286 (45.2)
Pervasive developmental disorders							
Autism	1,170 (0.1)	--	1,170 (10.6)		139 (0.1)	--	139 (22.0)
Other	331 (<0.1)	--	331 (3.0)		32 (<0.1)	--	32 (6.1)
Evaluation by surgeon, n (%)	293,855 (20.8)	291,550 (20.8)	2,305 (21.6)		--	--	--
Transplant received, n (%)	2,000 (9.4)	1,367 (12.8)	633 (5.9)[a]		--	--	--
Perioperative complication	--	--	--		30,750 (32.2)	30,549 (32.2)	201 (31.8)
90-day readmission	--	--	--		36,234 (37.9)	35,974 (37.9)	260 (41.1)
1-year mortality	--	--	--		1,200 (1.3)	N/A	N/A
Graft failure within 1 year	--	--	--		6,241 (6.5)	6,191 (6.5)	50 (7.9)
Graft rejection within 1 year	--	--	--		2,164 (2.3)	N/A	N/A

PC = population comparison group; IDD = intellectual or developmental disability group; IQR = interquartile range; CCI = Charlson comorbidity index;; N/A = Due to cell size restrictions in our data use agreement, these data cannot be presented

[a] n=3 kidney transplant recipients with IDD were unable to be matched

[b] These diagnoses are not mutually exclusive, so a single individual could be listed in multiple rows

© 2023 Hand BN et al. *JAMA Surgery.*
